# Safety and efficacy of vertebroplasty: Early results of a prospective one-year case series of osteoporosis patients in an academic high-volume center

**DOI:** 10.4103/0019-5413.53452

**Published:** 2009

**Authors:** Peter Diel, Dominique Merky, Christoph Röder, Albrecht Popp, Malgorzata Perler, Paul Ferdinand Heini

**Affiliations:** 1 Spine Service, Inselspital Bern, Switzerland; 2 Osteoporosis Policlinic, University Hospital, University of Bern, Switzerland; 3 Institute for Evaluative Research in Orthopedic Surgery, University of Bern, Bern, Switzerland

**Keywords:** Osteoporosis, percutaneous vertebroplasty, vertebral fractures

## Abstract

**Background:**

Vertebroplasty (VP) is a cost-efficient alternative to kyphoplasty. However, it is considered inferior when it comes to maintaining safety and in vertebral body (VB) height restoration. We assess the safety and efficacy of VP in alleviating pain, improving quality of life (QoL), and restoring alignment.

**Materials and Methods:**

In a prospective monocenter case series, from April 2007 until July 2008, 1,422 vertebroplasties were performed, during 307 interventions, in 279 patients with traumatic, lytic, and osteoporotic fractures with 28 repeat interventions, for new fractures after the primary surgery, in 28 patients. The 226 interventions (n=203 patients) done for osteoporotic fractures were analyzed for demographics, treatment and radiographic details, pain alleviation, QoL improvement [NASS and Euroqol (EQ-5D)] and complications.

**Results:**

Osteoporotic patient sample consisted of 77.8% (n=158) females with a median age of 78 years and 45 males who had the same median age. Around 69% of these patients were ASA (American Society of Anesthesiologists) grade 3 and above. On an average there were 1.8 VBs fractured and five VBs treated,whereas the most frequently performed cementations were in six (35.6%, n=80) or five (19.6%, n=44) levels. About 36.5%, (n=414) of the interventions were localized at the thoraco–lumbar junction (Th12–L2). On applying the Genant classification, there was a slight height reduction in 13.1% (n=29), a medium loss in 34.3% (n=78), and a severe loss of height in 52.6% (n=119). The pre-operative pain was assessed by the visual analog scale (VAS) and decreased from 56.7 to 41.4 pts after two months. Accordingly, the QoL on the EQ-5D measure (0.6 to 1) improved from 0.32 pts before surgery to 0.58 pts after two months. The pre-operative Beck index (anterior height/posterior height) improved from a mean of 0.66 preoperative to 0.80 post-operative and remained stable at two months post-operatively. There were cement leakages in 33% of the fractured VBs and in 0.8% of the prophylactically cemented VBs; there were symptoms in 7.1%, and most of them were temporary hypotension and one pulmonary cement embolism that remained asymptomatic.

**Conclusion:**

If routinely used, VP is a safe and efficient treatment option for osteoporotic vertebral fractures with regard to pain relief and improvement of the QoL. Even segmental re-alignment can be achieved to a certain extent with proper patient positioning.

## INTRODUCTION

A painful vertebral fracture can be a significant burden for patients, limiting physical function, quality of life (QoL) and increasing social isolation.^1^,^2^ Fractures may cause depression and can result in decreased mobility, loss of independence, and increased mortality because of a reduction in lung capacity and abdominal space with a consequent loss of appetite.^3^,^4^ In an osteoporotic population, the conservative treatment with bed rest, analgesics and orthotics leads to an additional loss of bone mineral density and muscle deconditioning, ending in a vicious cycle with increasing pain and additional fractures.^5^,^6^

Percutaneous vertebroplasty (VP) has been used in the treatment of osteoporotic compression fractures, aggressive hemangiomas and osteolytic neoplasms. Polymethylmethacrylate bone cement (PMMA) is injected into a fractured vertebral body (VB) through one or two bone biopsy needles.^3^ The cement is directly injected into the fractured vertebra without creation of a void unlike in balloon kyphoplasty (BKP). Therefore, higher pressure has to be applied, and cement leakage is more likely to occur. Moreover, re-establishment of the lost VB height is not possible, with the procedure per se, but can possibly be achieved with additional positioning maneuvers.^7^Height restoration, however, is not the main goal of VP but rather prevention of further segmental or spinal malalignment, pain reduction, increased mobility, and improved QoL.

The steady rise in the number of elderly people also results in a higher risk for vertebral fractures. The incidence (per 1,000 persons years) of vertebral fractures in the age group 50–54 years is 1.7 for males and 3.9 for females rising up to 14.6 and 25.7 in the age group 75–79 years.^8^ Therefore, treatment of vertebral fractures and the related costs become more and more important. In the United States (US), the total inflation adjusted, costs of VP rose from USD 76 million in 2001 to USD 152.3 million in 2005.^9^ In 1995, the direct expenditures for the treatment of osteoporotic fractures exceeded USD 13.8 billion in the US, and with the aging population, these costs are expected to increase up to USD 60 billion in 2030.^10^

The current article reports early results of 203 patients with one or several osteoporotic fractures in an academic center with a high annual volume of VPs.

## MATERIALS AND METHODS

Information was prospectively collected on standardized scanable case report forms in the framework of the research program for the treatment of osteoporotic fractures of the Association for the Study of Internal Fixation (AO/AO-ASIF). The data was then entered into the MEMdoc online database (www.memdoc.org) of the Institute for Evaluative Research in Orthopaedic Surgery (IEFO) at the University of Bern.^11^

The following documentation forms and outcome instruments were used: (a) surgeon-administered primary intervention form and follow-up form; (b) for patient assessment, Euroqol-5D, NASS, and comorbidity questionnaire; (c) patient consent form; and (d) one annotation form about the study and its purpose.

At the time of surgery, the primary intervention form is completed by the surgeon. Informed consent has to be given by the patient as a completed Euroqol-5D, NASS. Pre-operative comorbidity questionnaires have to be filled in at every follow-up examination after eight weeks, six months, one and two years..

The current article reports the two-month follow-up of the study. A total of 287 EQ-5D and 293 NASS forms for the evaluation of general and disease-specific QoL, and 151 comorbidity questionnaires were analyzed.

### Patient sample

#### Overall sample

In this prospective case series, 279 patients were treated, between May 2007 and July 2008, with a percutaneous VP. They underwent a total of 307 VP interventions with 1,422 treated levels. Twenty-eight repeat interventions were done, in 28 patients, for new fractures after primary surgery. Exclusion criteria for the study were - VP in combination with a rigid stabilization of the spine and a fracture older than six months or without reparative activity on MRI.

There were 196 (70.2%) females and 83 (29.8%) males with mean age of 74 years (range 28.4–94.1) and 71 years (range 34.9–92.7). The overall distribution of underlying diagnoses was osteoporosis in 72.7% (203 cases), trauma in 15.5% (43 cases), and lytic lesions in 11.8% (33 cases). Stratified by sex, there was osteoporosis in 80.6% (158 patients), trauma in 9.7% (19 patients), and lytic lesions in 9.7% (19 patients) of females. In the male patient group, there were 54.2% (45 patients) of cases with osteoporosis, 28.9% (24 patients) with trauma, and 16.9% (14 patients) of cases with lytic lesions.

The average number of cemented levels was 4.6. Stratified by diagnosis, about five levels were treated in osteoporotic patients. This included 2.9 levels in patients with trauma, and 4.3 levels in patients with tumors. The MRI was not routinely used to assess the fracture age, i.e., reparative activity. Only in those cases where multiple old and new fractures were present, the MRI and fracture edema were used in selecting the levels to be augmented.

The overall pre-operative ASA status (American Society of Anesthesiologists) of the patients was ASA 1 or 2 in 29.1% (88 interventions), ASA 3 or 4 in 63.3% (191 interventions), and there was one case (0.3%) with ASA 5. In 7.3% (22 cases), the ASA status was not recorded. Stratified by diagnosis, the following distribution was revealed: (a) osteoporosis: 24.8% (55 interventions) ASA 1/2, 67.7% (152 interventions) ASA 3/4, 6.8% (15 interventions) unspecified; (b) trauma: 54.8% (23 interventions) ASA 1/2, 35.7% (15 interventions) ASA 3/4, 2.4% (1 intervention) ASA 5, 7.1% (3 interventions) unspecified; (c) tumor: 26.3% (10 interventions) ASA 1/2, 63.2% (24 interventions) ASA 3/4, 10.5% (4 interventions) unspecified.

#### Study sample with osteoporosis

**Figure 1:**
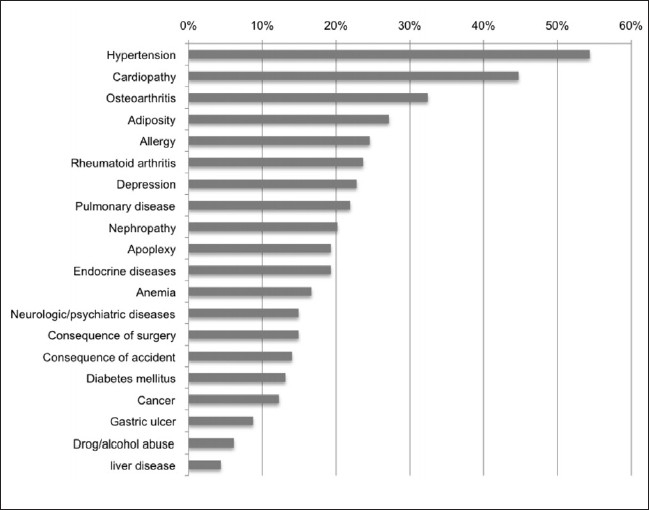
Comorbidities of the osteoporotic patient sample

The osteoporotic patient sample consisted of 77.8% (158) females, 45 males (22.2%) with a median age of 78 years. On an average, there were 1.8 VBs fractured. Osteoporosis was either defined based on dual axial absorptiometry (DXA) conducted during a current treatment in the hospital's Department of Osteoporosis (internally referred cases, about 50%) or based on anamnesis and risk/comorbidity profile of the patient. The main diagnosis could be specified as osteoporosis, trauma, or lytic lesion or a combination. For the current analysis, only cases with main diagnosis of osteoporosis were considered; this corresponds to a spontaneous or low-energy osteoporotic fracture. Cases where trauma and osteoporosis were marked, e.g., slipping in a bathtub with a consequent fracture, were excluded. The most frequently performed cementations were in 6 (35.6%, 80 interventions) or five (19.6%, 44 interventions) levels. About 36.4% (414) of the interventions were localized at the thoraco-lumbar junction, Th12–L2. Figure 1 shows the comorbidities of the osteoporotic patient sample.

#### Statistical analysis

Wilcoxon's rank-sum and signed-rank test were used for comparisons between baseline and follow-up examinations of continuous variables such as the pain visual analog scale (VAS). The chi-square test was used to compare proportions.

All statistical analyses were conducted using SAS 9.1 (SAS Institute Inc., Cary, NC), α was set to 0.05 throughout the study.

## RESULTS

### Pain relief

One of the main advantages of VP is fast and effective pain reduction. Pain was assessed by VAS scores using the NASS questionnaire. The mean pre-operative back pain was 56.7 points. At the eight-week follow-up, it was reduced to 41.4 points (*P* less than 0.0001).

### Reduction in pain medication

A significant reduction in pain killer consumption was revealed. The number of patients who did not need any pain medication increased from 8.4% (n=19) pre-operative to 46.2% (n=104) at the eight-week follow-up (*P* less than 0.0001). The number of patients consuming acetaminophen decreased, but not to a significant extent, from 40.1% (n=91) before intervention to 30.1% (n=68) (*P*=0.24). The consumption of metamizole decreased from 14.9% (n=34) pre-operative to 5.4% (n=12) at the eight-week follow-up time (p less than 0.005).

The consumption of non-steroidal anti-inflammatory drugs (NSAIDs) decreased insignificantly from 10.9% (n=25) before the intervention to 7.5% (n=17) at eight weeks postoperatively (*P*=0.15). Morphine and morphine derivates were needed by 25.8% (n=58) of patients before surgery. This number was reduced to 7.5% (n=17) at the eight-week follow-up time (*P* less than 0.004).

### Segmental kyphosis and alignment

For the evaluation of the segmental kyphosis and alignment, 137 patients whose radiographs were available for analysis at that point of time were radiologically assessed.

The average preoperative anterior VB height was 16.6 mm (range 31.4–5.2), improved postoperatively to 21.3 mm (range 7.4–32.4), and remained nearly stable after two months with an average of 21.2 mm (range 7.4–32.4).

The middle VB height was augmented from a preoperative average of 16.5 mm (range 5.3–27.1) to 20.2 mm postoperatively and after two months (range 9.1–29.2 and 10.8–29.6).

The average pre-operative Beck index (anterior height divided by posterior height) was 0.64 (range 0.19–1.58); the immediate post-operative one was 0.8 (range 0.29–1.25) and after two months, it remained stable at 0.8 (range 0.34–1.3) (*P* less than 0.0001).

The pre-operative local sagittal angle (angle of the superior and inferior end plate) was improved from an average 15.8° (range 0.4°–37.7°) to 9.4° (range 0.4°–30.6°) post-operatively and slightly worsened to 9.8° (range 0.1°–15.9°) after two months (*P* less than 0.0001).

### Fractured VBs—Genant classification

According to the Genant classification, out of 137 cases preoperative X-rays of which were available for analysis, no patient had a preoperative class 0 fracture; 13.1% (18 VBs) were class 1, 34.3% (47 VBs) class 2, and 52.6% (72 VBs) class 3. Post-operatively, out of 130 cases postoperative X-rays of which were available for analysis, 0.8% (1 VB) were class 0, 16.9% (22 VBs) class 1, 60 % (78 VBs) class 2, and 22.3% (29 VBs) class 3.

Two months post-operatively, there was no fracture class 0; 18.4% (18 VBs) were class 1, 66.3% (65 VBs) class 2, and 15.3% (15 VBs) class 3 (98 of 137 cases with available X-rays).

### QoL improvement

Possible values of the EQ-5D ranged from one (best possible QoL) to −0.6 (QoL worse than death). On a pre-operative examination, the mean EQ-5D score was 0.33 points. It improved to 0.58 points at the two-month follow-up (p<0.0001). Before the intervention, 24.2% (n= 49) of patients indicated a QoL below zero. At the two-month follow-up, this percentage was reduced to 3.2% (seven patients).

### Cemented levels—Fractured levels

**Figure 2:**
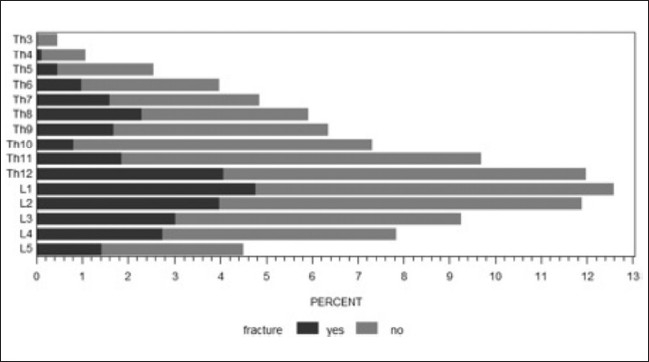
Frequency of cemented and fractured vertebral bodies

In total, 1,137 VBs were cemented in the group with osteoporosis as an underlying diagnosis. The most frequently treated levels were L1 in 12.6% (143), TH12 in 12% (136), and L2 in 11.9% (135) of cases. Of the 1,137 cemented levels, 364 had a fracture (32%). The most frequent fracture locations were L1 (15.7%, 57 cases), TH12 (13.7%, 50 cases), and L2 (12.9%, 47 cases). The other 773 levels (68%) were prophylactically cemented. Hence, with each fractured VB, about two others were prophylactically augmented [Figure 2].

### Subsequent fractures at the two-month follow-up

For risk assessment of new fracture risks, depending on the extent of preventive augmentation, we built four groups with patients who had been radiologically assessed or followed up by telephone. They were as follows: Group I (10 cases)—treatment of the fractured VB and preventive augmentation in not more than one level. There were 60% (six) women with an average age of 68 years. Males were on average 74 years old. Group II (38 cases)—multi-level prophylaxis either cranial or caudal to the fracture site. These were mostly cases with low lumbar or high thoracic fractures. There were 70% (27) women with an average age of 75 years. Males were 76 years of age. Group III (45 cases)—only adjacent cranial and caudal levels prophylactically cemented. There were 71% (32) females. Both sexes had an average age of 76 years. Group IV (92 cases)—multilevel, prophylaxis, cranial and caudal to the fracture site, there were 82% women; both sexes had an average age of 78 years. Two-month rates of new fractures in these four groups were 20, 6.7, 13.2, and 5.4%, respectively.

### Cement extrusions

**Figure 3:**
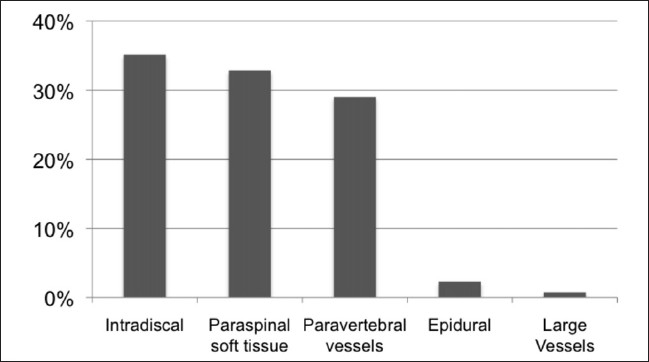
Direction of cement extrusions

There were 1,137 cemented VBs with 364 fractured and 773 prophylactically cemented levels in the osteoporotic patient sample. Overall, 126 (11.1%) cement extrusions were documented. For the fractured VBs, the extrusion rate was 33% (120/364) and 0.78% (6/773) for the prophylactically augmented VBs. The direction of extrusions is displayed in Figure 3.

Some authors seem not to consider the intra-discal extrusions as true extrusions or even provoke or undertake these “discoplasties” intentionally. If deducting this type of extrusion, the total rate of cement extrusions drops to seven per cent and respectively, for all fractured VBs to 20.3%. None of these extrusions caused radiculopathic symptoms.

### Intraoperative complications

Intra-operative complications were seen in 4.4% (9 cases). They were comprised of eight temporary hypotensions after cement injection (3.9%) and one cement embolism (0.5%).

### Post-operative experience of the patient with the procedure

At the two-month follow-up 72.9% of patients indicated that their condition felt “much” or “slightly” better; 7.6% considered the situation as “stable,”, 5.5% (5 cases) had declined, and 14% found the time too early to decide. A total of 80% of the patients would undergo the same operation getting the same result “certainly” or “probably”; 17% were not sure and only 3% would “probably” or “certainly” not undergo the operation again.

## DISCUSSION

Our series reports short-term results of the treatment of VB compression fractures with per-cutaneous VP in osteoporotic patients. We found a significant and clinically relevant reduction in back pain, decreased pain killer consumption, increased QoL, and vertebral height restoration. The underlying disease was osteoporosis, a condition with a reduced bone density and limited skeletal stability. The socio-economic interest is increasing with the rising prevalence of osteoporosis in the aging population.

Osteoporotic vertebral fractures are associated with reduced QoL, limited physical function, and increased risk for further fractures, and they predict the total mortality.^12^,^13^

VP is an accepted minimal-invasive treatment for these fractures. In the past few years, a large number of studies have shown the safety, efficacy, and cost-effectiveness of this treatment.^14^–^20^

This study has showed moderate cement extrusion rates but much lower symptomatic extrusions compared with the literature. The intra-operative hypotension was observed as patients were monitored very closely and the intervention was done in general anesthesia. No clinically symptomatic cement leakages were observed in this series.^21^–^25^,^14^ An additional augmentation of adjacent and nonadjacent VBs was used to prevent further fractures and all the related consequences, to minimize the total number of surgeries in this multi-morbid patient population, and to consequently increase the cost-effectiveness of the index intervention.^26^–^28^ The decision to perform a multilevel preventive augmentation was based on patient's age, gender, comorbidity risk profile (e.g., renal disease, steroid treatment), previous osteoporotic VB fractures, previous osteoporotic fractures in other bones, and the number and location of newly fractured VB. Therefore, the long-term results of this case series will be illuminative in showing if there is a valid correlation between the number of prophylactically treated levels and the rate of new fractures in the osteoporotic spine. The current short-term analysis of new fractures at the eight-week follow-up in the four groups showed the highest proportion of new fractures in group I with a maximum of one preventive augmentation and the lowest in group IV with cranial and caudal multilevel augmentation, despite the highest percentage of females and the oldest average age of patients in this group. In the literature, the rate for distant or adjacent fractures is between 17% and 27% depending on the follow-up time.^27^,^28^,^29^ Unfortunately, there is no objective parameter yet which is helpful for assessing the individual fracture risk of the most vulnerable levels.

A significant improvement in the anterior and/or central VB height could be shown. This was also reflected in a significant pre- to post-operative improvement in the Beck index. Percutaneous VP and BKP, both have the ability to restore the vertebral height and to improve the alignment.^30^ For VP, however, the pre-operative dynamic mobility of the fracture is the important predictor for the postoperative height improvement which is mainly achieved by a correct prone positioning maneuver and not by the procedure itself.^31^

A significant back pain reduction from 56.7, preoperatively, to 41.4 at the eight-week follow-up was found. The pain reduction might appear limited but it is still significant and clinically relevant. However, it also reflects the fact that we are inclined to a rather aggressive approach for the treatment of osteoporotic VB compression fractures in order to prevent further collapses. Therefore, we consider the intervention as indicated even in cases with an initially moderate pain level. The pain alleviation, reduced need for medication, and improved segmental alignment increased the QoL after VP to a great extent.

The significant and clinically relevant pain relief we found is also reported by other authors in observational study designs.^32^,^33^ Randomized controlled trials are needed for a conclusive evaluation of pain relief in VP, BKP and conservative treatment programs..

In meta-analysis and systematic reviews of the literature, a significantly greater improvement in pain scores was found in patients receiving VP.^34^ There was, however, no difference in the clinical significance of pain relief between the two treatments. In comparison to the conservative treatment regimens, both VP and BKP are promising innovations with the benefit of rapidly improved mobility, function, stature, significantly decreased pain-related doctor visits, and reduced use of analgetics.^35^,^36^

## CONCLUSION

VP results in immediate back pain reduction as well as improvement of local sagittal alignment, compared to baseline. Along with it goes an improved QoL and low rates of complications and revisions, compared to baseline. The number of preventive augmentations reduced the short-term new-fracture rate in this osteoporotic patient sample without significantly increasing the complication and extrusion rates.
